# Evaluation of Static Displacement Based on Ambient Vibration for Bridge Safety Management

**DOI:** 10.3390/s24206557

**Published:** 2024-10-11

**Authors:** Sang-Hyuk Oh, Hyun-Joong Kim, Kwan-Soo Park, Jeong-Dae Kim

**Affiliations:** 1 Hybrid Structural Testing Center, Myongji University, 116, Myongi-ro, Yongin-si 17058, Republic of Korea; oshmju@mju.ac.kr; 2 NOW E&S Co., Ltd., 20-20, Techno valley 1-ro, Jillye-myeon, Gimhae-si 50875, Republic of Korea; nowens2023@naver.com (K.-S.P.); now2947@naver.com (J.-D.K.)

**Keywords:** aged bridge, displacement, modal analysis, ambient vibration, wireless accelerometer

## Abstract

The evaluation of bridge safety is closely related to structural stiffness, with dynamic characteristics and displacement being key indicators. Displacement is a significant factor as it is a physical phenomenon that bridge users can directly perceive. However, accurately measuring displacement generally necessitates the installation of displacement meters within the bridge substructure and conducting load tests that require traffic closure, which can be cumbersome. This paper proposes a novel method that uses wireless accelerometers to measure ambient vibration data from bridges, extracts mode shapes and natural frequencies through the time domain decomposition (TDD) technique, and estimates static displacement under specific loads using the flexibility matrix. A field test on a 442.0 m cable-stayed bridge was conducted to verify the proposed method. The estimated displacement was compared with the actual displacement measured by a laser displacement sensor, resulting in an error rate of 3.58%. Additionally, an analysis of the accuracy of displacement estimation based on the number of measurement points indicated that securing at least seven measurement points keeps the error rate within 5%. This study could be effective for evaluating the safety of bridges in environments where load testing is difficult or for bridges that require periodic dynamic characteristics and displacement analysis due to repetitive vibrations, and it is expected to be applicable to various types of bridge structures.

## 1. Introduction

The aging of infrastructure, particularly bridges, has become a major concern worldwide. Most bridges were constructed in the 1960s, and considering their typical lifespan of about 50 years, they are now entering a significant stage of aging. This trend is expected to accelerate sharply. In the United States, many infrastructures built in the 1930s began to show signs of aging by the 1980s, highlighting the need for maintenance and management. However, due to budget constraints, appropriate repairs and reinforcements were not carried out. As a result, collapse incidents occurred, such as the I-35W Mississippi River Bridge in Minnesota, which was completed in 1967 and collapsed in 2007; the I-5 Highway Bridge in Washington, completed in 1955 and collapsed in 2013; and more recently, the Fern Hollow Bridge in Pittsburgh, completed in 1973 and collapsed in 2022, resulting in injuries to over ten people. These incidents have significantly increased the demand for budget expansion and market interest in aging infrastructure.

In South Korea, approximately half of the 30,000 bridges were constructed around the year 2000. Among them, 5662 bridges, accounting for 17.9% of the total, are over 30 years old. This percentage is expected to surge to 49.7% in the next decade [1]. The Jeongja Bridge, built in 1993, collapsed in 2023, resulting in two casualties and further highlighting the urgent need for improved maintenance and management measures for aging bridges. In evaluating the safety of such aging bridges, the dynamic characteristics and displacement of bridges are crucial indicators. Particularly, displacement is a key measurement item for evaluating the safety of bridges as it not only demonstrates the structural behavior characteristics directly related to the stiffness of the bridge but also is a physical phenomenon that bridge users can directly perceive [2].

A common method for measuring bridge displacement is to install LVDT and conduct vehicle load tests, applying loads to the bridge and measuring the resulting displacement. While LVDT offers high precision, it requires a fixed reference point for measuring displacement. Securing a work platform or wires for the fixed reference point not only consumes significant time and cost but also may be difficult in certain situations. A typical example is when there are obstacles under the bridge, such as rivers, oceans, roads, or railways. Additionally, in cases where the space beneath the bridge is very high, the work platform and wires used for displacement measurement may be affected by wind-induced vibrations, leading to significant measurement errors. Furthermore, the high-altitude work poses safety risks for workers. Consequently, various studies have been conducted on techniques for analyzing dynamic characteristics and displacement to evaluate the safety of bridges where direct displacement measurement is difficult and where existing technologies are challenging to apply due to cost, traffic volume, and environmental impacts. However, due to the high costs of these technologies and the need for specialized personnel, their practical application is extremely limited, with most cases remaining at the research stage.

Jeon and Lee proposed an algorithm to estimate displacement based on the relationship between strain and analytical displacement. While this method is based on a simple theory that defines the relationship between strain and displacement through structural analysis and estimates displacement from measured strain, for accurate analysis, synchronization between the analytical results and the test results must be achieved. Additionally, attaching strain gauges under the bridge still has the disadvantage of installation difficulty depending on the environment beneath the bridge [3].

An algorithm was implemented to measure bridge displacement data using accelerometers and strain gauges, allowing for the calculation of structural displacement. This method effectively prevents low-frequency loss found in existing acceleration-based displacement estimation techniques and analyzes the displacement of bridges. However, the installation of strain gauges is essential, and vehicle load testing is still required, which remains a limitation [4,5,6,7].

A displacement estimation method using ambient vibration was proposed to overcome the challenges of traditional load testing. This method extracts the dynamic characteristics of the bridge from ambient vibrations, improves the initial finite element model, and uses the improved model to numerically calculate displacement, thus estimating the displacement correction factors that can be obtained through traditional load testing. However, this method is difficult to generalize due to the wide range of variables that users can select for model improvement. Considering the need for skilled experts and the cost of structural analysis, it may not be suitable for general bridges but is considered more appropriate for the maintenance systems of large bridges [8].

In addition, displacement measurement systems such as image-based displacement, laser, and GNSS require the installation of targets, which are affected by the environment beneath the bridge [9,10], and due to the high cost of the equipment, they are not suitable for use in bridge safety diagnosis tests. The technology proposed in this paper involves installing accelerometers on the upper part of the bridge and estimating displacement based on ambient vibrations. It is not affected by the environmental conditions beneath the bridge, and load testing is unnecessary, making it significantly more efficient and practically applicable compared with existing technologies.

This paper proposes a technique for estimating the dynamic characteristics and displacement, which are key indicators for evaluating the safety of bridges, using ambient vibrations without the need for load testing. The proposed technique was applied to an actual bridge to verify its accuracy. This method involves extracting mode shapes and natural frequencies from ambient vibration data measured using wireless accelerometers and then analyzing the flexibility matrix to estimate displacement at specific locations.

The proposed method enables the easy and economical analysis of displacement in bridges where the installation of displacement meters is challenging. It is expected to effectively evaluate the safety and usability of various types of bridges, including not only road bridges but also railway bridges that experience periodic vibrations. Importantly, as it can be developed into an automated long-term maintenance system rather than being limited to one-time load testing, further research is anticipated to facilitate the expansion of its applicability.

## 2. Theoretical Background Ambient Vibration Analysis

### 2.1. TDD Technique

The time domain decomposition (TDD) technique is based on the principle that spatial variables, such as mode shapes, and temporal variables, such as natural frequencies and damping ratios, can be independently separated and extracted. This method directly extracts mode shapes in the time domain without Fourier transformation for the frequency range where natural frequencies exist, allowing for the procedures for extracting natural frequencies and mode shapes to be performed independently. Thus, the TDD technique is particularly suitable for extracting mode shapes in large structures that require many sensors [11,12,13].

Additionally, the TDD technique uses a digital band-pass filter to obtain the time response of a single mode from acceleration signals instead of using the inverse Fourier transform of the frequency domain decomposition (FDD) technique or the random decrement (RD) function of the Ibrahim time domain (ITD) technique. This approach does not require advanced data processing techniques and can be implemented with simple programs, making it easier to handle large amounts of real-time data in automated system processing. Furthermore, this single-degree-of-freedom approach is known to avoid the occurrence of spurious modes that can arise in the eigensystem realization algorithm with data correlation (ERADC) and ITD techniques [14,15,16].

**Figure 1:**
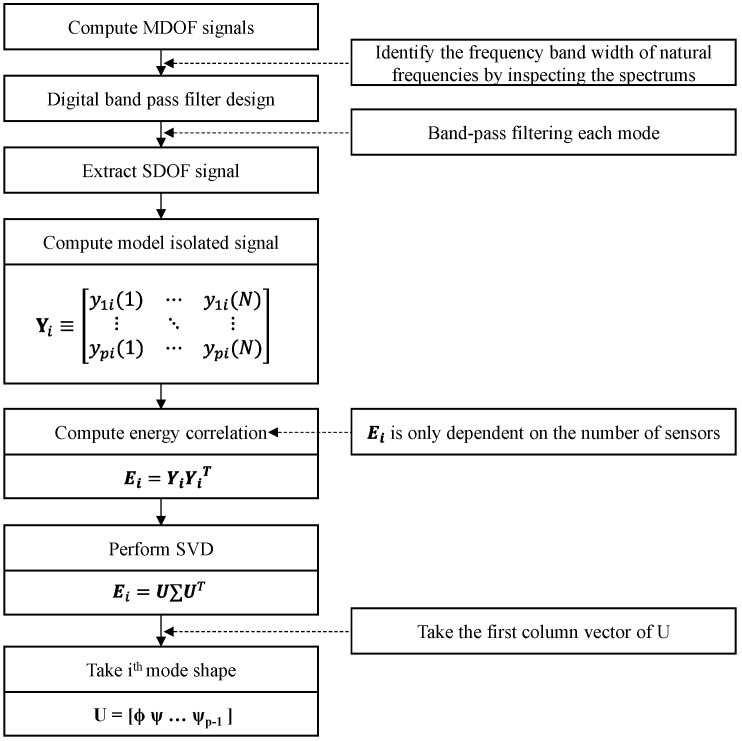
TDD technique for mode shape extraction.

The TDD technique is performed according to the procedure shown in Figure 1. First, using digital band-pass filters, the ambient vibration accelerations measured by p accelerometers are processed to calculate the time response data matrix $Y_{i}$ that contains only the frequency components expected to include the $i^{th}$ natural frequency. If N time samples are measured using p accelerometers, the size of $Y_{i}$ is p×N times. Second, the energy correlation matrix $E_{i}$ is defined as shown in Equation (1).

(1) $$E_{i}=Y_{i}Y_{i}^{T}$$

The singular value decomposition (SVD) of the energy correlation matrix $E_{i}$ is performed as shown in Equation (2).

(2) $$E_{i}=U\sum U^{T}$$

In Equation (2), $U=[\varphi_{i},{\;\psi }_{1},\;\ldots ,\;\psi_{p-1}]$ represents the singular vector matrix and $\sum =[q_{i},\;\sigma_{i},\;\ldots ,\;\sigma_{p-1}]\;$represents the singular value matrix. $\psi_{i}$ in the singular vector matrix represents the noise mode, while $q_{i}$ in the singular value matrix represents the intensity of the dominant mode, and $\sigma_{i}$ represents the energy of the noise mode. Because the dominant energy magnitude in the $i^{th}$ single-degree-of-freedom (SDOF) acceleration response is represented by the $i^{th}\;$mode shape $\varphi_{i}$, the singular values are ordered as $q_{i}>\sigma_{i}>\cdots >\sigma_{p-1}$. Therefore, the $i^{th}$ mode shape in Equation (2) corresponds to the first column vector of the singular vector matrix U of $E_{i}$ [11]. The size of the energy correlation matrix $E_{i}$ is p×p, and it is not a function of the number of time samples N, but rather a function of the number of sensors p. Therefore, it is not proportional to the number of measured time samples N, significantly reducing the amount of computation required for singular value extraction, making it suitable for real-time automation. This characteristic makes the TDD technique suitable for the real-time extraction of mode shapes in large structures, such as cable-stayed bridges, which require the installation of numerous sensors.

### 2.2. Extraction of Natural Frequencies

The cross-correlation function $c_{i}\left(k\right)\;$representing the $i^{th}$ mode can be obtained using the $i^{th}$ mode shape extracted through the space-time partitioning method, as shown in Equation (3). Here, $y\left(k\right)$ is a p×1 vector representing the $k^{th}$ measured acceleration matrix sample.

(3) $$c_{i}\left(k\right)=\frac{\emptyset_{i}^{T}}{\emptyset_{i}^{T}\emptyset_{i}}y(k)$$

Because this cross-correlation function vector is the single-degree-of-freedom time response filtered by the $i^{th}$ mode, it has the same form as the free vibration function with the $i^{th}$ mode shown in Equation (4) [17].

(4) $$c\left(k\right)=Ae^{{-\xi }_{i}\omega_{i}k∆t}cos(\omega_{d}k∆t-\theta )$$

where A is the amplitude, $\omega_{i}$ is the undamped natural frequency, $\xi_{i}$ is the damping ratio, $\omega_{d}$ is the damped natural frequency ($\omega_{i}\sqrt{1-\xi^{2}}$), and θ is the phase angle. The parameters to be identified in Equation (4) are the natural frequency, damping ratio, amplitude, and phase angle. The natural frequency ω of the $i^{th}$ mode can be extracted by applying the sensitivity-based system identification (SI) method as an extension of the time domain decomposition (TDD) technique [18]. The SI method, a type of inverse analysis, optimizes the variables until the rate of change of the identified variables becomes zero by iterating until the measured and simulated values are equal. Thus, the SI method inevitably involves a repetitive process, requiring extensive calculations, making it challenging to implement automated programs.

In this paper, a simple and rapid method was applied to extract the natural frequency without iterative processes. It can be observed that three consecutive acceleration cross-correlation time samples are closely related for a uniform sampling time ∆t.

(5) $${a_{0}c_{i}\left(k\right)+a}_{1}c_{i}\left(k+1\right)=\;c_{i}\left(k+2\right)\;\;\;,\;\;\;k=0,1,\cdots ,N-2$$

The unknowns $a_{0}$ and $a_{1}$ are functions of the $i^{th}$ natural frequency $\omega_{i}$ and damping ratio $\xi_{i}$ as follows:

(6) $$a_{0}=-e^{-\xi_{i}\omega_{i}2\Delta t}$$

(7) $$a_{1}=2e^{-\xi_{i}\omega_{i}\Delta t}cos\left(\omega_{d}\Delta t\right)$$

Applying the measured acceleration cross-correlation function $c_{i}\left(k\right)$ in Equations (3)–(5) generates an over-determined system (m × n) as follows:

(8) Ax=b

where $A=\left[\begin{array}{cc} c_{i}\left(0\right) & c_{i}\left(1\right)\\ \vdots & \vdots \\ c_{i}\left(N-2\right) & c_{i}\left(N-1\right) \end{array}\right]$,$b=\left[\begin{array}{c} c_{i}\left(2\right)\\ \vdots \\ c_{i}\left(N\right) \end{array}\right]$, $x=\left[\begin{array}{c} a_{0}\\ a_{1} \end{array}\right]$.

The least squares method can be used to find the approximate solution to Equation (8).

(9) $$x={\left(A^{T}A\right)}^{-1}A^{T}b$$

Once the coefficients $a_{0}$ and $a_{1}$ in the x term are obtained from Equation (9), the natural frequency $\omega_{i}$ of the $i^{th}\;$mode can be calculated using the system of Equations (6) and (7).

### 2.3. Flexibility Matrix

The flexibility matrix is the inverse of the global stiffness matrix. An approximation of the flexibility matrix obtained by combining modal parameters is called the dynamic flexibility matrix. The $j^{th}$ column vector of the dynamic flexibility matrix represents the displacement vector when a unit load is applied to the $j^{th}$ degree of freedom. In other words, the approximate $j^{th}$ flexibility row vector obtained by combining modal parameters is called the $j^{th}$ dynamic flexibility vector. The $j^{th}$ dynamic flexibility vector, $f_{j}$, can be obtained as a linear combination of modal parameter terms using Equation (10):

(10) $$f_{j}=\sum_{i=1}^{r}\frac{\phi_{ji}}{\omega_{i}^{2}m_{i}}\phi_{i}$$

where the p×1 vector $\phi_{i}$ represents the $i^{th}$ mode shape normalized to have a maximum value of 1, and the variable $\phi_{ji}$ represents the value at the $j^{th}$ element of the $i^{th}$ mode shape vector $\phi_{i}$. The variable r represents the total number of modes considered. The variables $\omega_{i}$ and $m_{i}$ represent the $i^{th}$ natural frequency and modal mass, respectively. The $i^{th}$ modal mass can be approximated using Equation (11):

(11) $$m_{i}=\rho A\int_{0}^{L}\phi_{i}(x)\phi_{i}(x)dx$$

where the variables ρ and A represent the mass and cross-sectional area of the bridge, respectively. The mass per unit length, ρA, can be calculated from the design documents or by using a finite element model to derive the average cross-sectional area.

The relationship between the $i^{th}$ mode shape vector $\phi_{i}$ is normalized to have a maximum value of 1, and the mode shape vector $\psi_{i}$ is normalized by the modal mass $\phi_{i}$ = $\sqrt{m_{i}}\psi_{i}$ [19]. Therefore, the $j^{th}$ dynamic flexibility given in Equation (12) can also be expressed as:

(12) $$f_{j}=\sum_{i=1}^{r}\frac{\psi_{ji}}{\omega_{i}^{2}}\psi_{i}$$

As shown in Equation (12), as the $i^{th}$ natural frequency $\omega_{i}$ is in the denominator, the contribution of higher modes to the flexibility is small, while the contribution of lower modes is significant. The dynamic flexibility obtained from the above Equation represents the displacement at each node (acceleration measurement point) of the bridge when a 1 N load is applied. That is, by applying vehicle loads, design loads, etc., to the unit load displacement at the point where the maximum displacement of the bridge occurs, the static displacement of the bridge can be predicted without actual load application.

**Figure 2:**
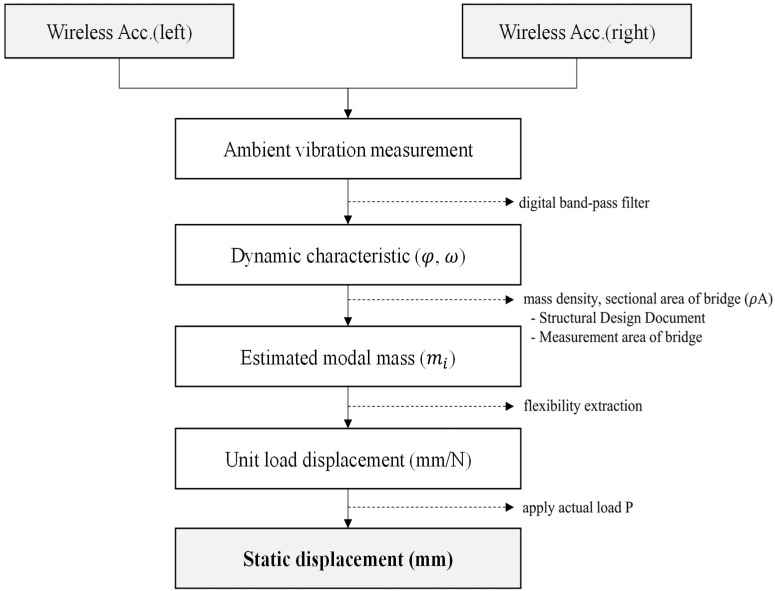
Flowchart of bridge static displacement estimation using ambient vibration.

The proposed method for estimating bridge displacement using ambient vibration is summarized in the flowchart shown in Figure 2. Wireless accelerometers are installed at equal intervals on both sides of the bridge to measure ambient vibrations. The reason for installing them on both sides is to exclude torsional modes in the vertical direction of the bridge and extract only the bending modes through a modal shape analysis. Using the measured ambient vibrations, the modal shapes and natural frequencies are calculated based on the TDD technique. The flexibility curve is then derived by calculating the mass per unit length of the bridge through as-built documents or structural analysis. The extracted flexibility curve can be considered as the displacement caused by a force of 1 N, and by applying the actual load, the static displacement of the bridge can be estimated. This method can be applied as an effective maintenance system for bridges where it is difficult to perform vehicle load testing, as well as for railway bridges where constant vibrations occur repeatedly, as it allows for the prediction of displacement based on stiffness without the need to apply actual loads to the bridge.

## 3. Verification by Field Test

### 3.1. Outline of Field Test

**Figure 3:**
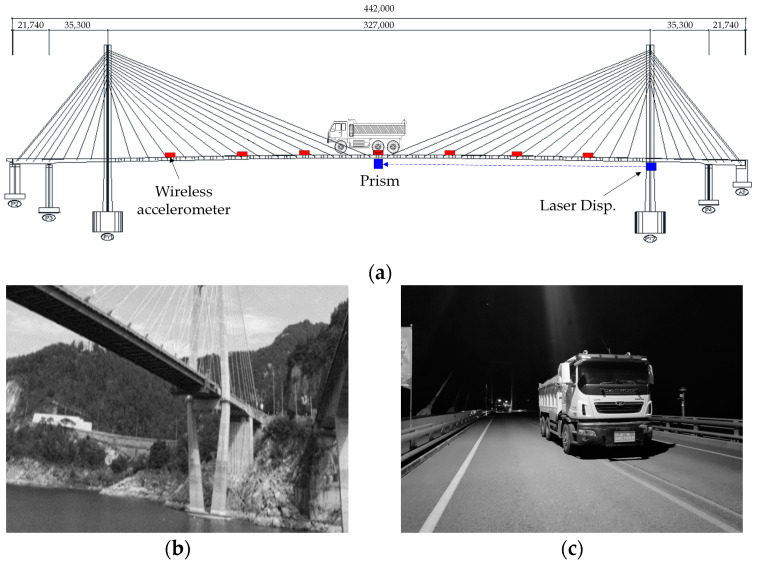
Overall view of the test bridge: (**a**) a drawing of the test bridge; (**b**) bridge substructure; (**c**) static load testing.

**Figure 4:**
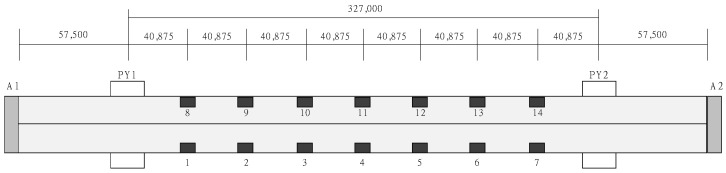
Wireless accelerometer installation location.

To verify the field applicability of the proposed method, a demonstration test was conducted on an in-service bridge. As shown in Figure 3a, the test bridge is a two-tower cable-stayed bridge with a total span of 442.0 m and a width of 13.0 m, completed in September 2010. Figure 3b shows the superstructure and substructure views of the bridge. Because there is a lake beneath the bridge, it is not possible to apply the conventional method of installing LVDTs on the substructure to measure displacement. To measure the displacement at the midspan, where the maximum displacement occurs, accelerometers were installed in the main span (327.0 m), and ambient vibration monitoring and loading tests were conducted. As shown in Figure 3a and Figure 4, the wireless accelerometers were installed at 7 points on both sides of the bridge at equal intervals of 40.875 m, resulting in a total of 14 points for ambient vibration measurements. The ambient vibration measurements were conducted during the daytime, when there was heavy traffic, to ensure sufficient data collection. The measurements were taken for approximately 1 h and 30 min at a sampling frequency of 128 Hz, focusing on the vertical acceleration component. The data obtained from the ambient vibration measurements included not only the dynamic behavior of the bridge but also environmental noise (such as wind, vehicles, and external vibrations). However, by collecting data over an extended period, such noise can be averaged or canceled out, allowing for a clearer extraction of the bridge’s inherent dynamic characteristics.

The detailed specifications of the wireless accelerometers used in the test are shown in Table 1. For the ambient vibration measurement of the test bridge, the G-Link-200 wireless accelerometers from M company were used. The wireless accelerometers have a resolution of 20 bits, equivalent to approximately 120 dB in the dynamic range, allowing for the precise measurement of both large and small vibration variations under various environmental conditions. When multiple sensors are installed on the bridge and measurements are taken at sampling frequencies above 100 Hz, if the time is not synchronized, distortions in the dynamic behavior analysis of the structure may occur, leading to errors in the analysis of mode shapes and natural frequencies and impacting the accuracy of displacement estimation. Each sensor was synchronized within ±50 microseconds based on the server time, and data transmission was managed through allocated time slots and the data buffer function to prevent any data loss.

**Figure 5:**
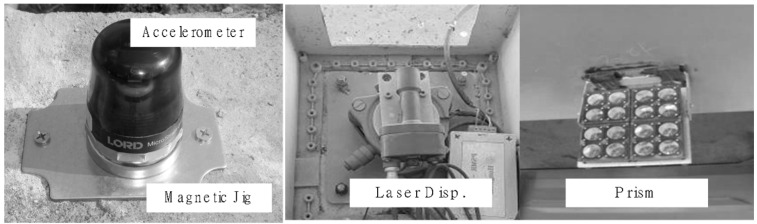
Accelerometer and laser displacement sensor for bridge response measurement.

Meanwhile, to measure the actual displacement of the bridge, a load test was conducted using a dump truck with a total weight of 270 kN, fully loaded with soil. After completely restricting traffic, the truck was positioned at the center of the bridge for a static load test to apply the maximum load, as shown in Figure 3a,c. The test followed the guidelines in the Load Test Manual for the Safety and Maintenance of Bridges in Korea, positioning the rear wheel center of the vehicle at the location of the bridge’s maximum moment. The displacement values obtained from the static load test were measured using the laser displacement sensor (PSM-R) of the bridge’s maintenance monitoring system. The PSM-R is a long-distance displacement measurement sensor capable of measuring up to 400 m, and it monitors the vertical displacement of the bridge 24 h a day with a sampling rate of 20 samples per second. The detailed specifications of the laser displacement sensor used in the test are shown in Table 2. The laser displacement sensor was installed at PY2, with the prism target located at the midpoint of the bridge. The wireless accelerometers, a laser displacement sensor, and a prism target installed on the test bridge are as shown in Figure 5.

The bridge analysis model referred to the MIDAS/Civil structural analysis data from the detailed safety inspection in 2023, and the analytical results of the natural frequencies were compared with the results of the continuous vibration measurements.

### 3.2. Dynamic Characteristic Analysis

The most common method used for bridge dynamic characteristic analysis, the PP (peak picking) method, involves converting acceleration signals collected in the time domain into the frequency domain via Fourier transform to extract natural frequencies. While this method is simple to apply, it is influenced by the resolution of the frequency domain. Additionally, mode shape analysis requires calculating the transfer function, phase function, and coherence function, making real-time processing and mode shape implementation challenging for large structures with many sensors [20]. The TDD (time domain decomposition) method used in this study, on the other hand, first extracts mode shapes, which are spatial variables, in the frequency domain and then extracts the corresponding time variables. This method involves simpler calculations compared with traditional methods and can be implemented with simple software, enabling immediate analysis, even in the field.

**Figure 6:**
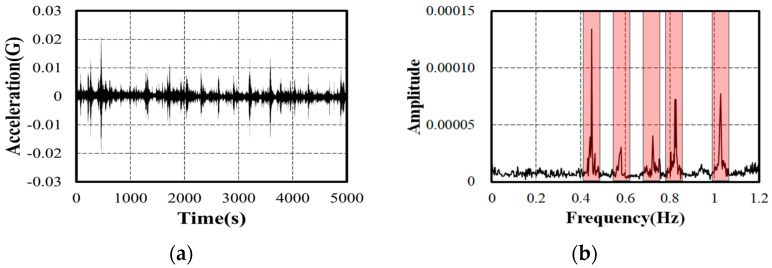
Inspection of the measured signal: (**a**) acceleration time response from sensor 4; (**b**) FFT result.

Figure 6a shows the ambient vibration data measured from sensor 4 located at the center of the bridge. To calculate the power spectrum of the data measured by each sensor, the NFFT was set to 8192 points, resulting in a frequency resolution of 0.015625 Hz. The Hanning window, known to be most suitable for continuous waveform analysis, was applied. After calculating the power spectrum for the acceleration signals measured at each point, the approximate frequency bands for each mode were selected, as shown in Figure 6b. Two peaks were identified in the frequency range of 0.4~0.6 Hz, with additional peaks observed near 0.7 Hz, 0.8 Hz, and 1.0 Hz. A digital band-pass filter was selected to extract single-degree-of-freedom signals for the selected frequency bands. The frequency response of the test bridge shows that the excited major mode frequencies exhibit clear peaks, indicating a lightly damped system. In a lightly damped system, even a low-order band-pass filter shows sufficient effectiveness. Furthermore, using a high-order filter increases the computational load and can cause Gibbs phenomena. Therefore, a third-order Butterworth filter, known for having minimal ripple among digital band-pass filters, was used [16].

**Figure 7:**
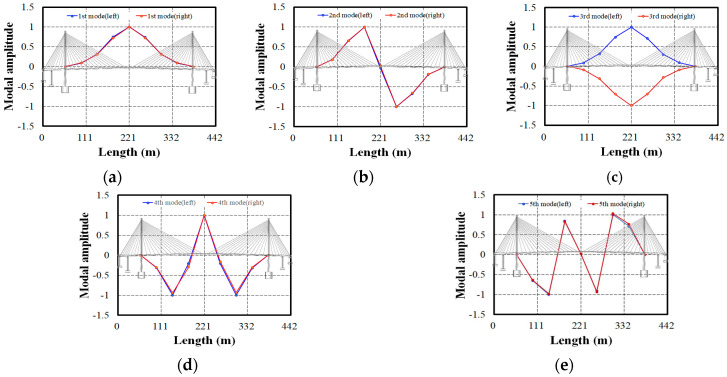
The extracted mode shapes using the TDD technique: (**a**) 1st mode; (**b**) 2nd mode; (**c**) 3rd mode; (**d**) 4th mode; (**e**) 5th mode.

The single-degree-of-freedom signals ($Y_{i}$) extracted using the digital filter were used to obtain the energy correlation matrix $E_{i}$ from Equation (1). Finally, the singular value decomposition (SVD) of $E_{i}$ was performed to extract the mode shapes from the singular vector U. It is important to note that the energy correlation matrix $E_{i}$ for the singular value analysis, calculated for each single-degree-of-freedom signal, is solely a spatial domain variable, excluding the time variable. Although the number of time samples is 691,200, only 14 measurement points need to be considered. In contrast, traditional methods such as ERADC, ITD, or FDD require the matrix size for a singular value analysis to be a function of both the number of measurement points and time samples. Thus, as the number of measurement points and measurement time increases, the computational load grows exponentially. However, as the TDD method is a function of the measurement points only, large mode shapes can be extracted in real time. Four bending and one torsion mode shapes, extracted from the test bridge, are shown in Figure 7. The test bridge’s mode shapes in the vertical directions appear almost identical, indicating no significant stiffness difference between the left and right sides of the bridge. This suggests that the bridge exhibits stable behavior without any left–right stiffness differences, and it can be further inferred that the cable tension is also behaving stably.

The mode shapes of long-span bridges and large structures requiring vibration management are used as fundamental data for understanding the smooth behavior at support points, estimating damage, and improving complete numerical models. For long-span bridges, where dynamic behavior is crucial, extracting high-resolution mode shapes is essential for the maintenance of the structure. Traditional modal analysis techniques face an exponential increase in the size of data matrices and the computational load for complex operations like singular value decomposition (SVD) as the number of measurement points increases. In contrast, the TDD (time domain decomposition) method is highly efficient for extracting multi-point high-resolution mode shapes of large structures because the increase in numerical computations is linear with the increase in measurement points.

**Figure 8:**
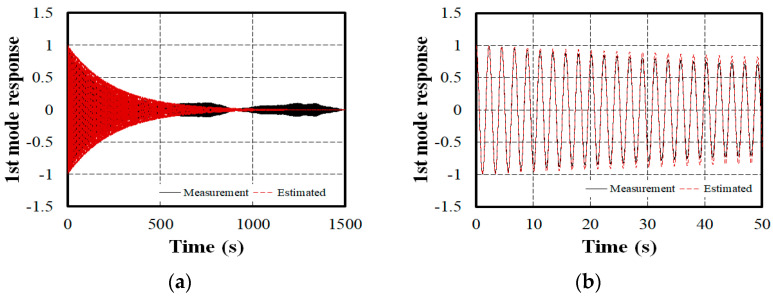
Comparison of the estimated model and measured cross-correlation of the 1st bending mode in the Z dir.: (**a**) t = 1500 s; (**b**) t = 50 s.

To extract the natural frequencies of the bridge, the cross-correlation functions representing each mode were calculated using the five mode shapes obtained earlier and using Equation (3), and the natural frequencies were extracted using Equation (5). Figure 8a shows the cross-correlation acceleration signal of the first mode extracted. The measured cross-correlation signal exhibited noise after approximately 500 s, so the natural frequencies were extracted from the signal up to t = 500 s. The fundamental assumption is that the extracted cross-correlation function resembles the free vibration function with the $i^{th}$ mode as it is a single-degree-of-freedom time response. By substituting the time variables, such as the extracted natural frequency and damping ratio for the first mode, into Equation (4) and comparing it with the first mode’s cross-correlation function obtained from Equation (5), as shown in Figure 8b, the period and damping magnitude match well. This indicates that the natural frequencies and damping ratios extracted using the proposed method are highly reliable.

Table 3 shows the natural frequencies of the bridge identified for each mode order. The final extracted natural frequencies of the bending modes from the first to the fourth order were 0.4477, 0.5785, 0.8256, and 1.0271 Hz, respectively. The torsional mode’s first-order frequency was identified as 0.7185 Hz. The error rates compared with the analytical values were 0.6067%, 2.5709%, 1.0549%, 0.9291%, and 0.1365%. Generally, when the measured natural frequencies of a bridge are higher than the design values, it suggests that there has been no decrease in stiffness, indicating that the structural safety margins remain sufficient.

### 3.3. Displacement Analysis

The mode shapes and natural frequencies of the vertical bending modes extracted by the TDD method, along with the mode mass, enable the derivation of the displacement curve under unit loading. The mode mass for each order can be approximately calculated using Equation (11). The mass per unit length of the bridge (ρA) can be determined by calculating the average cross-sectional area from the design documents or using the analytical model. The weight per unit length of the test bridge was referenced from the structural calculation report in the completion documents, and as shown in Table 4, the weight per unit length of 146.066 kN/m was confirmed through the cross-sectional areas and unit weights of each member.

The modal mass was calculated using the unit mass of the bridge, mode shapes, and Equation (11), as shown in Table 5. The mode contribution at position j for the $i^{th}$ mode can be calculated using the calculated modal mass of the $i^{th}$ mode, the value of position j for the $i^{th}$ mode, and the natural frequency of the $i^{th}$ mode.

**Figure 9:**
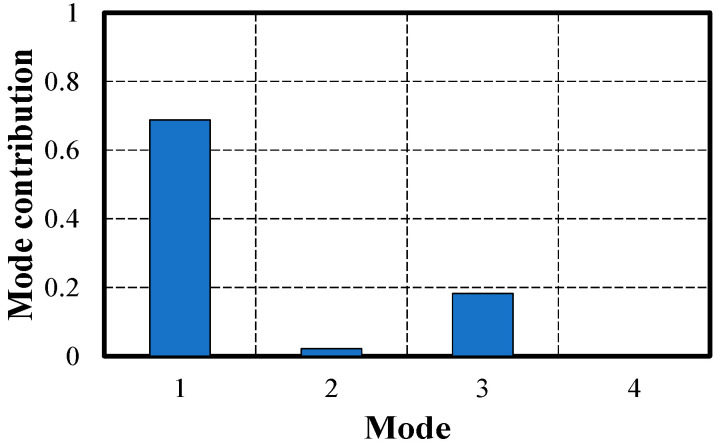
The contribution of modes on modal flexibility at the midspan.

The modal contributions to the flexibility at the midspan of the bridge, as shown in Figure 9, indicate that the first mode is the most dominant. The contributions beyond the fourth mode are minimal. This represents the displacement contribution of each mode at the midspan, suggesting that for the displacement estimation at the center of the bridge, considering up to the fourth bending mode is sufficient.

**Figure 10:**
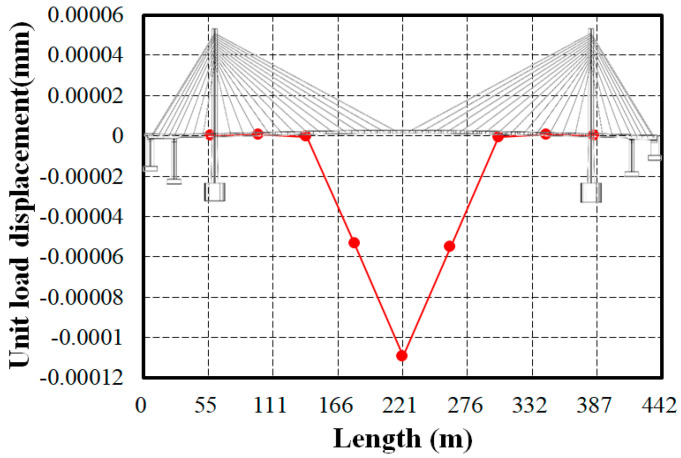
Estimating the flexibility for the Z dir. load of 1N at 221.0 m.

The displacement curve when a unit load (1N) is applied at the midspan of the bridge is shown in Figure 10. The estimated displacement at this point is −1.09 × 10^−4^ mm. The overall shape of the displacement curve indicates that the maximum displacement occurs at the center of the bridge, with almost no displacement near the pylons. This tendency is also observed in the first mode shape, which had the most dominant influence, likely due to the high tension maintained in the short length stay cables near the pylons. However, detailed investigations into the causes and relationships will require further research into the relationship between cable tension and displacement.

**Figure 11:**
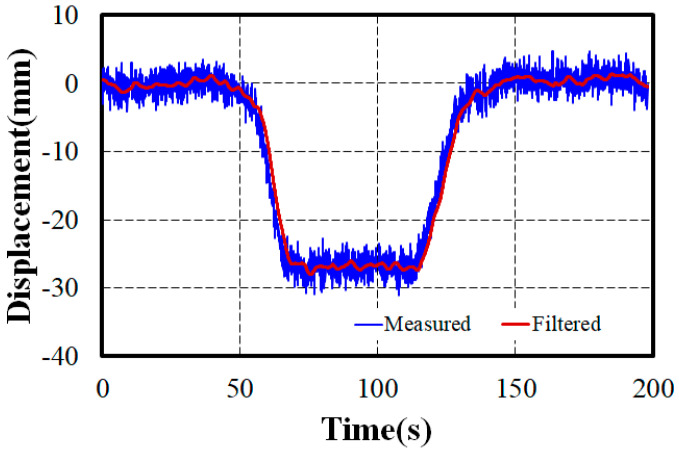
Measured displacement using a laser sensor for a load of 270 kN by load testing.

Assuming a linear load–displacement relationship for the bridge, the static displacement can be estimated by applying an arbitrary load P to the unit load displacement curve analysis results. The unit load displacement at the midspan, where the maximum displacement occurs, was −1.09 × 10^−4^ mm. When applying the load of 270 kN from the load test dump truck, a displacement of −29.49 mm was estimated. Meanwhile, the laser displacement sensor measurements from the static load test, shown in Figure 11, include noise and disturbances. This indicates that the measurement contains dynamic components due to the bridge’s inherent vibrations and the inherent noise of the laser displacement sensor, implying some inherent error. This noise was reduced by using a third-order Butterworth low-pass filter with a cutoff frequency of 0.2 to remove high-frequency components and emphasize low-frequency components. The filtered signal indicates a displacement of approximately −28.47 mm for the applied load of 270 kN.

**Figure 12:**
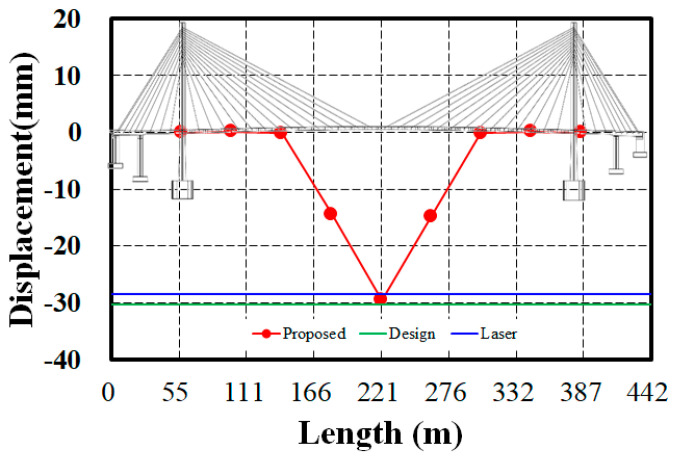
Comparison of the estimated, measured, and design displacement at the midspan for 270 kN.

The estimated static displacement using the proposed method, the actual displacement measured by the laser displacement sensor, and the displacement from the structural analysis are compared in Figure 12. The structural analysis displacement is the theoretical value from the design at the time of completion, used to assess the bridge’s safety by determining whether the measured displacement exceeds the design theoretical value. The estimated displacement from the proposed method is −29.49 mm, the actual measured displacement by the laser displacement sensor is −28.47 mm, and the analytical value is −30.21 mm. Because these values do not exceed the theoretical value, it indicates that the bridge’s stiffness and displacement behavior remain stable compared with the time of completion. This is consistent with the results obtained from the stiffness comparison using natural frequencies. The difference between the estimated displacement and the measured displacement is 1.02 mm, showing an error rate of approximately 3.58%. Increasing the number of wireless accelerometers to enhance the resolution of the mode shapes may reduce the error rate, but considering the minimal contribution of modes beyond the fourth mode and the practicality and cost-effectiveness of fieldwork, the current level of displacement accuracy is deemed adequate.

### 3.4. Effect of Number of Measurement Points

The number of continuous vibration measurement points is the most critical factor influencing the accuracy of mode shape extraction, which forms the basis of the proposed method. Increasing the number of continuous vibration measurement points can contribute to higher resolution mode shape extraction and thus greater accuracy in displacement analysis. However, considering test costs and work efficiency, this study aimed to analyze the optimal number of sensors that would be advantageous for practical applications.

**Figure 13:**
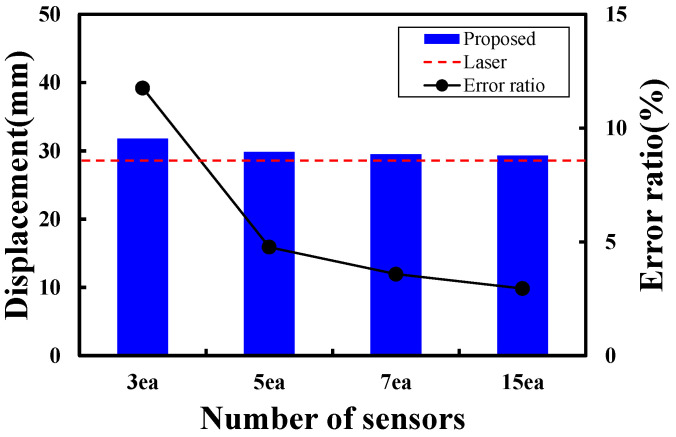
Error rate of the estimated displacement according to the number of measurement points.

Wireless accelerometers were installed at the main span of the test bridge in quantities of 3, 5, 7, and 15 to measure continuous vibrations. The displacement estimation results for each configuration are compared in Figure 13. As the number of measurement points decreased from 15 to 3, the midspan displacement increased to −29.31 mm, −29.49 mm, −29.83 mm, and −30.82 mm, respectively. Comparing these with the actual measured value of −28.47 mm obtained from the laser displacement sensor, the estimated displacement using 15 sensors exhibited an error rate of 2.95%, while configurations with seven and five sensors showed error rates of 3.58% and 4.77%, respectively, all within a 5% error range. In contrast, the configuration with only three sensors demonstrated a significantly reduced accuracy with an error rate of 11.76%. This substantial decrease in accuracy is attributed to the limited mode shape extraction and greatly diminished resolution when only three sensors are employed. Furthermore, the estimated displacement of −30.82 mm exceeds the design value of −30.21 mm, potentially leading to a misinterpretation that there is an issue with the stiffness of the bridge. For small bridges, where displacements are typically within approximately 5 mm, an error margin exceeding 5% renders the analysis results unreliable [21]. As shown in the error rate graph by the number of sensors in Figure 13, convergence begins with the use of seven sensors, and the difference compared with using 15 sensors is minimal, within about 1%. Therefore, to extract up to the third mode, which has a significant contribution, and to ensure an error rate of within 5% compared with the actual measurements from the laser displacement sensor, it is considered necessary to secure at least seven measurement points for continuous vibration measurements.

## 4. Conclusions

In this study, a new method for estimating bridge displacement using continuous vibration data was proposed and validated. The method involves extracting mode shapes and natural frequencies from continuous vibration data measured by wireless accelerometers and estimating static displacement under arbitrary loads using a flexibility matrix. Field validation on an actual cable-stayed bridge demonstrated that the estimated displacement using the proposed method showed a high accuracy with an error rate of 3.58% compared with displacements measured using a laser displacement sensor. The main findings and conclusions of this study are as follows:

1. Ambient vibration data of the bridge were collected using wireless accelerometers, and mode shapes and natural frequencies were extracted using the TDD method. By utilizing the extracted mode shapes and natural frequencies, a flexibility analysis based on the bridge’s stiffness was conducted to calculate the unit load displacement (mm/N), representing the combined contribution of each mode to the bridge’s displacement. By applying an arbitrary load P to this calculated unit load displacement, it was possible to evaluate the static displacement of the bridge under various loads without performing actual load tests.
2. The field test results indicated that the unit load displacement was −1.09 × 10^−4^ mm. When applying a vehicle load of 270 kN, the estimated displacement was −29.49 mm. This showed a 3.58% error compared with the actual measured displacement of −28.47 mm obtained from the laser displacement sensor, and it was less than the structural analysis value of −30.21 mm, confirming no concerns about the reduced stiffness of the bridge. This result demonstrated the feasibility of applying the developed method for structural condition assessment based on stiffness and displacement, especially for small bridges where load tests are not feasible and for bridges requiring continuous vibration monitoring.
3. Analyzing the accuracy of displacement estimation based on the number of continuous vibration measurement points, the study found an error rate of 11.76% with 3 measurement points and 2.95% with 15 points. Although increasing the number of measurement points improved the accuracy of displacement estimation, considering the field applicability and cost-effectiveness of the proposed method, securing seven measurement points, where the error converges, is deemed appropriate.
4. The use of continuous vibration measurements with wireless accelerometers and flexibility analysis achieved a displacement analysis accuracy within 5% compared with traditional methods. However, the proposed method requires additional field validation for complex bridge structures such as arch bridges or variable cross-section bridges. Given that the current detailed safety inspections for bridge safety assessment include load-bearing capacity evaluations, further research is needed to develop techniques for estimating load-bearing capacity factors, such as impact factor estimation.
5. Although the unit load displacement is calculated based on the actual stiffness of the bridge, the stage of evaluating static displacement by applying an actual load P assumes a linear load–displacement relationship. Generally, bridges exhibit linear behavior within small load ranges, but non-linear behavior can occur as the load increases. Complex bridge structures distribute and transfer loads through various paths, which can accentuate non-linearity. Therefore, additional laboratory tests and field validations on various bridge types are necessary to accurately understand the actual behavior of bridges and improve the accuracy of displacement evaluation. Further detailed research considering structural characteristics and loading conditions should be conducted.

The proposed method enables the analysis of the dynamic characteristics and displacement of bridges using only ambient vibrations, making it simpler and more economical than the traditional method of installing displacement sensors and conducting vehicle load tests to measure bridge displacement. Furthermore, with the advancement of this method, it is expected to be highly useful as a long-term maintenance monitoring system for railway bridges, where vibrations occur periodically, and for structures vulnerable to vibrations.

## Figures and Tables

**Table 1:** The specification of the wireless accelerometer G-Link-200.

Specification	
Measurement range	±2 G, ±4 G, ±8 G configurable
Integrated sensors	Triaxial MEMS accelerometer, 3 channels
Resolution	20 bit
Sampling mode	Continuous, periodic burst, event triggered
Sampling rate	1 Sample/hour to 4096 Hz
Node synchronization	16 M bytes (up to 8,000,000 data points)
Wireless communication range	Outdoor/line-of-sight: 800 m
Radio frequency	License-free 2.405 to 2.480 GHz with 16 channels
Power source	3 × 3.6 V, 1/2 AA batteries
Compatible gateways	WSDA gateway

**Table 2:** The specification of the laser displacement sensor PSM-R.

Specification	
Measurement distance	10~250 m
Resolution	0.001% RO (<50 m), 0.0027% RO
Reflector	Prism (<50 m), prism group (>50 m)
Sampling rate	20 Hz

**Table 3:** Extracted of natural frequency by ambient vibration measurement.

Mode	Frequency (Hz)		Remark
	Proposed	Design	
1st	0.4477	0.4450	1st bending
2nd	0.5785	0.5640	2nd bending
3rd	0.7185	0.7110	1st torsion
4th	0.8256	0.8180	3rd bending
5th	1.0271	1.0257	4th bending

**Table 4:** Calculation of weight per unit length of the bridge.

Member Load	m^2^	kN/m^3^	kN/m
Deck	3.72	25.0	93.000
Steel girder	0.135	78.5	13.738
Floor beam	0.046	78.5	4.71
Stringer	0.018	78.5	1.413
Roadway pavement	0.8	23.0	18.400
Sidewalk pavement	0.075	23.0	1.725
Handrail	-	-	2.000
Handrail base	-	-	11.080
Total	-	-	146.066

**Table 5:** Calculation of the modal mass for each bending mode.

Mode	Hz	kg
1st bending	0.4477	1.29 × 10^6^
2nd bending	0.5785	1.45 × 10^6^
3rd bending	0.8256	1.44 × 10^6^
4th bending	1.0271	1.72 × 10^6^

## Data Availability

Data are contained within the article.
